# Temporal control of i-motif switch lifetimes for autonomous operation of transient DNA nanostructures†
†Electronic supplementary information (ESI) available: Detailed experimental procedures, materials and methods, a list of all oligonucleotide sequences and supporting figures. See DOI: 10.1039/c7sc00646b



**DOI:** 10.1039/c7sc00646b

**Published:** 2017-03-31

**Authors:** L. Heinen, A. Walther

**Affiliations:** a Institute for Macromolecular Chemistry , University of Freiburg , Stefan-Meier-Str. 31 , 79104 Freiburg , Germany . Email: andreas.walther@makro.uni-freiburg.de; b Freiburg Materials Research Center , University of Freiburg , Stefan-Meier-Str. 21 , 79104 Freiburg , Germany; c Freiburg Center for Interactive Materials and Bioinspired Technologies , University of Freiburg , Georges-Köhler-Allee 105 , 79110 Freiburg , Germany

## Abstract

Functional DNA nanotechnology creates increasingly complex behaviors useful for sensing, actuation or computation, as enabled *via* the integration of dynamic and responsive structural DNA motifs. However, temporally controlled and dynamic DNA structures with programmable lifetimes, that are able to operate autonomously and self-revert to the starting state are challenging to achieve due to tedious and very system-specific sequence design. Here, we present a straightforward concept to program transient lifetimes into DNA duplexes based on the pH-sensitive DNA i-motif switch. We integrate the i-motif switch with an internal, non-linear pH-resetting function using a rationally designed chemical reaction framework, by which the switch autonomously undergoes a complete “off–on–off”-cycle without the use of additional external triggers. The lifetime of the activated “on”-state (*i.e.* the hybridized state) can be systematically programmed over several hours. The system can be readily implemented into hybrid DNA structures on larger length scales. Focusing on autonomous materials, we demonstrate temporal control of transient fluorescence signals and temporary aggregation of gold nanoparticles.

## Abstract 2

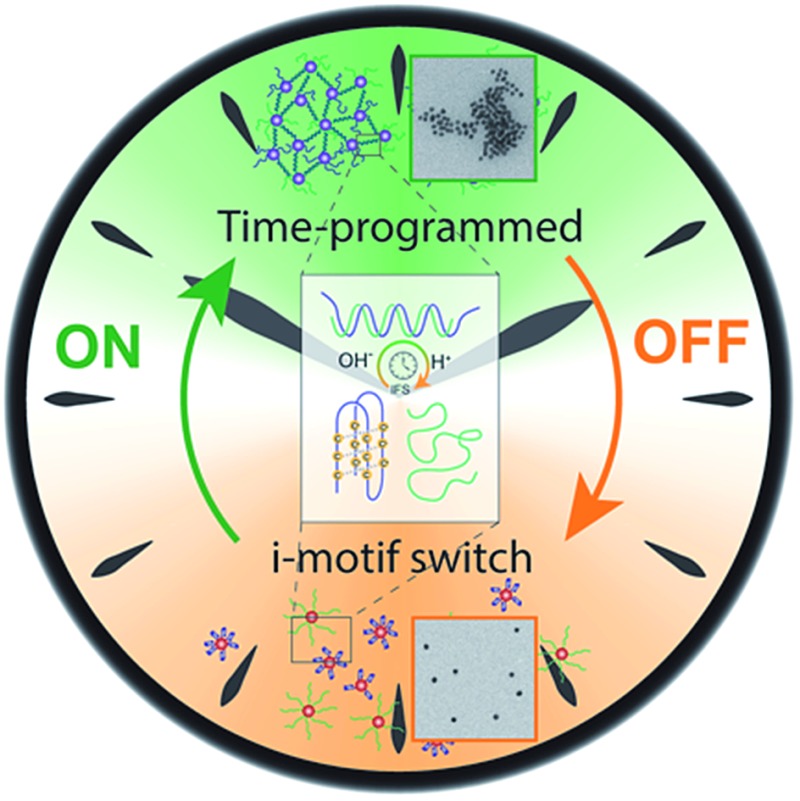
System integration of the DNA i-motif switch with a tunable pH environment allows programmable lifetimes of DNA duplex hybridization and higher level self-assemblies in closed and autonomous systems.

## Introduction

The sequence-specific base pairing of oligonucleotides permits precise molecular programming of DNA self-assemblies. Over the last decades this knowledge has led to increasingly complex synthetic DNA structures with regard to spatial construction and functionality, as shown, *e.g.*, by the assembly of hierarchical architectures based on DNA origami or the creation of nanodevices that are able to perform work or locomotion as well as tasks like sensing, reporting or computing *via* logic gates.^1–10^ The integration of switchable DNA sequences has allowed progress from purely static to dynamic structures that respond to certain stimuli and environmental changes *e.g.*, small molecules, DNA oligonucleotides/fuel strands, temperature, pH, light, ionic strength.^11,12^

Among those stimuli, in particular pH-sensitive motifs, such as the i-motif or triplex structure, have come into focus, as they can be switched easily and offer access to new types of materials.^13,14^ The i-motif, a cytosine-rich sequence, has emerged as a versatile pH-switchable sequence for functional dynamic DNA structures (more structural details below). For instance, it has proven useful as a spatiotemporal pH indicator inside living cells, for on-demand activation of DNAzymes, pH-driven DNA walkers, rotors and tweezers, reorganization of origami tiles, logic operations and switchable material properties (*e.g.* surface hydrophilicity) or pH-triggered DNA hydrogels with shape-memory effect.^15–26^

These examples illustrate the reliable and robust character of the i-motif sequence, making it highly attractive for use in dynamic structures. Nonetheless, the aforementioned systems lack autonomy, and changes rely on manual triggering of pH changes by direct addition of acid or base. System control has been improved by coupling the pH switching to indirect drivers, such as pH-oscillators, a photobase and enzymatic reactions.^21,27–31^ Yet, these approaches provide autonomous temporal operation of the dynamic switching of the i-motif only to a very limited extent in fully closed systems and need still interference from the outside for reliable and precise temporal control.

True dynamic behavior is more complex and exceeds simple responsiveness and reversibility as it takes place under energy dissipation to afford transient metastable states.^32^ To this end, dynamic structures require a fuel to operate in an autonomous fashion temporarily out of equilibrium. Currently, most operation mechanisms of dynamic DNA structures rely on toehold-mediated strand displacement technology, which is enabled either by sequential addition of fuel/instruction strands, or more intricately, by cascaded chain hybridization reactions to drive *e.g.*, DNA walkers and motors.^4,33,34^ However, these structures typically run down on the global energy ladder from one initial state to a final low energy state. An autonomous return to the initial state has remained difficult to achieve. Therefore, dynamic DNA circuits, such as oscillators and autonomous DNA motors usually include enzymatic degradation of certain strands to escape this thermodynamic deadlock.^35–38^

Another alternative has been the development of catalytic fuel strands, which allows repeated cycling of dynamic hybridization cascades.^39–42^ Therein, control over the kinetics of competing strand-displacement reactions is the key for continuous and resettable dynamic operation cycles, which requires elaborate sequence design.^43^

Here, we present a system approach to impose a temporally controlled transient lifetime on the pH-sensitive i-motif switch. The system operates dynamically in closed systems in a self-regulated fashion without the need of external intervention after an initial starting point. Conceptionally, we pursue a strategy towards autonomous dynamic DNA structures by pushing the environment of the i-motif switch temporarily out of equilibrium *via* a kinetically controlled dynamic transient pH change.^44^ The i-motif switch is coupled to this internal pH feedback system and responds with temporary activation. We show how to program the timescale of this internal pH resetting mechanism, and how this allows to program the lifetime of the activated i-motif switch (*i.e.*, hybridized duplex).

Our focus is on temporal programming of autonomously reversible DNA (hybrid) structures to create active material properties and functions, as being demonstrated by transient fluorescence signals and gold nanoparticle aggregation. This approach demonstrates the first example of a transient and programmable lifetime of pH-regulated DNA switches, and opens the design space for straightforward temporal programming of DNA structures with more complex, autonomous and time-dependent behaviors.

## Results and discussions

### Characterization of the pH-responsive i-motif switch

**Fig. 1:**
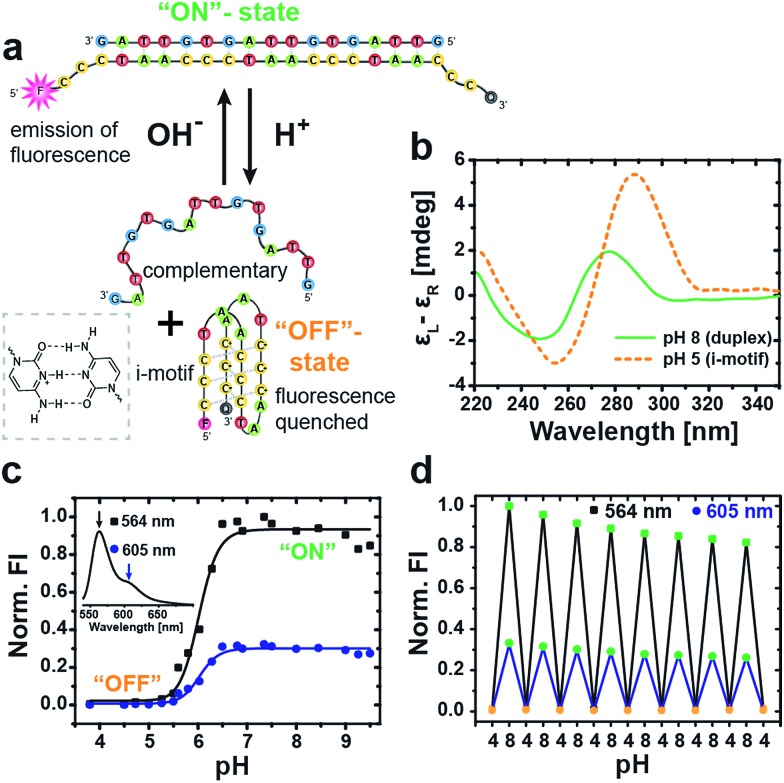
Characterization of the i-motif switch under different static pH conditions. (a) Schematic illustration of the two conformational states of the i-motif switch with the closed hybridized “on”-state (duplex) at alkaline pH and the open “off”-state (i-motif + random coil) at acidic pH. The i-motif tetraplex is stabilized by hemiprotonated cytosine^+^–H–cytosine base pairs (left box). The i-motif switch is modified with a Cy3 fluorophore (F) and a Black Hole Quencher 2 (Q) to produce fluorescence upon photoexcitation (*λ*_exc_ = 520 nm) as output signal for the active “on”-state at high pH. (b) CD spectra of the unlabeled switch show the characteristic bands of duplex B-DNA at pH 8 and of the folded i-motif at pH 5. (c) Fluorescence intensity (FI, normalized to maximum) of the i-motif switch in dependence of pH evaluated at the two maxima of the Cy3 fluorophore at 564 nm (black squares) and 605 nm (blue dots). Inset shows the full emission spectrum of Cy3 for the activated i-motif switch (“on”-state). (d) Multiple cycling between acidic and alkaline pH switches the fluorescence reversibly “on” and “off”. Slight dilution effects due to addition of acid and base are visible.

The basic DNA component for our autonomous DNA systems is the i-motif switch, a so-called proton-fueled DNA nanomachine.^15^ The classic i-motif is characterized by an intramolecularly folded tetrameric structure formed by cytosine-rich sequences at slightly acidic pH. Partial protonation of the cytosine nucleobases leads to the stabilization of parallel duplexes *via* hemiprotonated cytosine^+^–H–cytosine base pairs. Two parallel duplexes then associate and interdigitate in an antiparallel manner to give rise to the i-motif tetraplex (Fig. 1a).^45,46^

Our DNA i-motif switch comprises the cytosine-rich i-motif strand (I), and another complementary strand (C) with two intentional mismatches (Table SI1† lists all oligonucleotide sequences).^15^ The two mismatches prevent strand C from folding into a G-quadruplex, and, further, they lower the melting temperature of the duplex. The switch adopts two different states depending on the pH: at alkaline conditions, both strands hybridize into a rigid duplex (active closed state = “on”-state, pH 8), whereas, at slightly acidic pH, the cytosine nucleobases get partially protonated, inducing the formation of the i-motif tetraplex. As a result, the switch opens and separates into two parts yielding the folded i-motif tetraplex (I) and the complementary strand (C) in a random coil conformation (open state = “off”-state, pH 5).

The two distinct conformational states of the switch can be identified by circular dichroism (CD) spectroscopy (Fig. 1b). At pH 8, the CD spectrum shows the characteristics of B-DNA (duplex) with a positive band at 278 nm, a crossover at 262 nm and a negative band of minor intensity at 248 nm. The open state is revealed at pH 5 *via* a pronounced positive band at 289 nm, a crossover at 270 nm and a negative band at 255 nm, being indicative of the folded i-motif structure.^15^

For a more straightforward *in situ* characterization of the switching behavior, we equipped the i-motif strand (I*) with the pH-insensitive^47^ fluorescent cyanine dye Cy3 (F) at the 5′-end and the Black Hole Quencher 2 (Q) at the 3′-end.

By this means, fluorescence emission is solely turned “on” under alkaline conditions when the switch is closed by hybridization with the complementary (C) strand, which spatially separates the fluorophore/quencher pair. In contrast, the fluorescence is quenched in the “off”-state at acidic pH through efficient FRET (Förster resonance energy transfer) between the dye and the quencher due to their close proximity in the folded conformation. The full fluorescence spectrum of the closed switch at pH 8 is depicted in the inset of Fig. 1c, showing a strong emission peak of Cy3 at 564 nm with a minor shoulder at 605 nm.

An evaluation of the pH-dependent fluorescence intensity (FI) at both wavelengths allows to determine the transition of the i-motif switch from the “off” to the active “on”-state at the sharp increase between pH 5.5 and 6.5 with a sigmoidal fit to a transition midpoint of pH 6.0 (Fig. 1c).

Molecular reversibility of the switch is profoundly important for generating a time-programmed function with a transient active “on”-state, as it is required for complete deactivation with restoration of the initial “off”-state. We therefore performed multiple cycling between pH 4 and pH 8 by alternating addition of 1 M NaOH_(aq)_ or 1 M HCl_(aq)_, and the concurrent switching between low and high FI demonstrates excellent reversibility of the i-motif switch (Fig. 1d). The slight, progressive decrease in FI arises from dilution.

### Temporal control of i-motif switch lifetimes *via* integration with an internal pH feedback system

Next, we integrate the i-motif switch with a pH-feedback system allowing to achieve temporary “on”-switching in a closed system by decoupling kinetically the activation and deactivation, that is the deprotonation and protonation reaction of the cytosine bases, respectively. The kinetic boundary condition for realizing a transient assembly of the switch requires (i) faster deprotonation than protonation (*v*_act_ ≫ *v*_deact_) for successful activation, and (ii) that the activation caused by deprotonation ceases with time (*v*_act_ → 0) to give way to an effectively delayed deactivation. We master this kinetic constraint by installing an internal pH feedback system (IFS) that creates a dynamic pH loop from acidic to basic to acidic conditions, to which the i-motif switch responds with transient hybridization into the “on”-state.

**Fig. 2:**
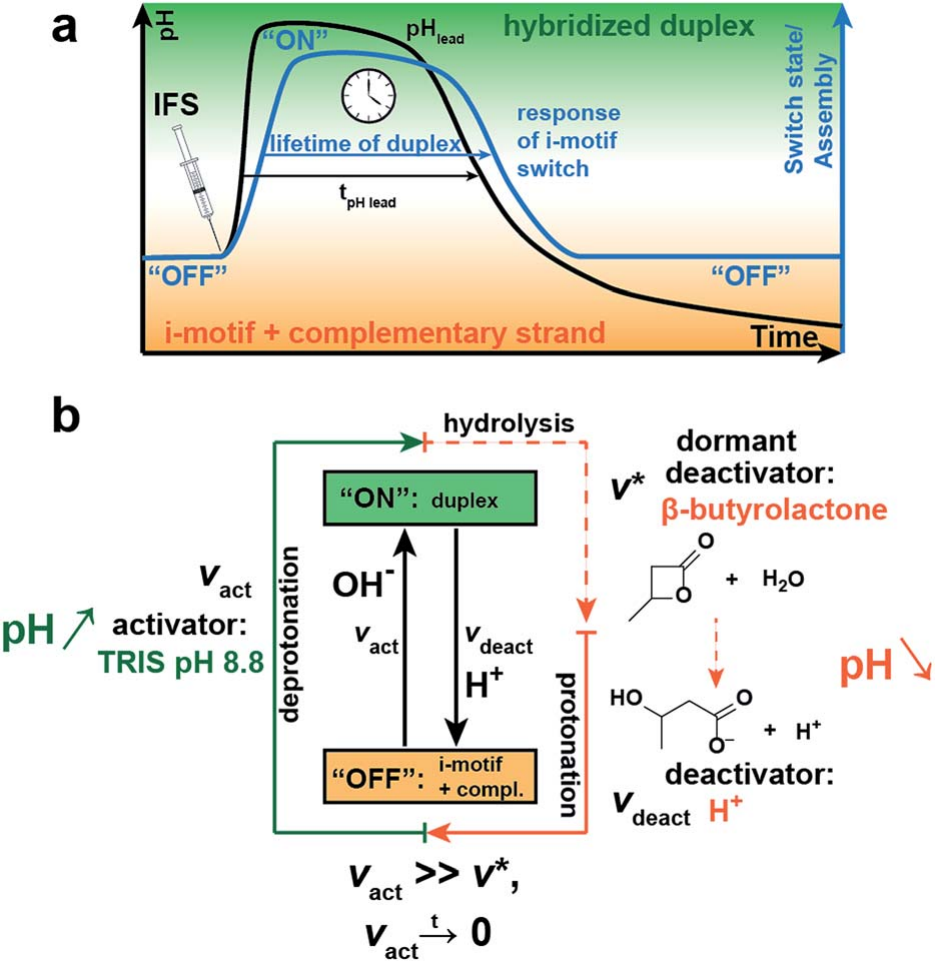
Concept for temporal programming of the pH-sensitive i-motif switch by an internal resetting mechanism. Installation of an internal pH feedback system (IFS), composed of a fast activator (Tris pH 8.8) and a dormant deactivator (DD, β-butyrolactone), creates a transient alkaline pH state. (a) Simultaneous injection of both compounds leads to an instant rise of pH with a delayed pH decay due to slow hydrolysis of the DD (black line) and concurrent formation of the deactivating acid. The i-motif switch couples to the IFS and responds with temporary “off–on–off” switching (blue line). (b) Kinetic conditions for self-regulated transient activation of the i-motif switch: the activation step *v*_act_ (deprotonation) needs to be much faster than the deactivation step *v*_deact_ (protonation) and eventually ceases with time. This is realized by the slow hydrolysis rate v* of the abundant DD to the true deactivator: *v*_act_ ≫ v*, *v*_act_ → 0.

The IFS is based on a combination of a fast activator and a dormant deactivator (Fig. 2a).^44^ The activator in the form of an alkaline buffer substance initiates an immediate pH rise (fast *v*_act_) to an alkaline pH plateau. Concurrently, the dormant deactivator (DD), a hydrolytically unstable ester compound, forms acid (H^+^) as the true deactivator. This occurs in a delayed fashion due to an inherent slow hydrolysis rate (v*), which is given by the molecular structure of the ester, and is pH-dependent and autocatalytic in its nature.^44^ This lowers the pH slowly and decouples by this means activation and deactivation in time (*v*_act_ ≫ v*). Owing to the dormant character of the deactivator, both components of the IFS can be injected simultaneously without instantaneous compensation of these two opposing cues, which would be impossible when directly using acid and base. This allows temporal programming in closed systems and application as an internal resetting mechanism. Importantly, both triggers are strongly coupled as the hydrolysis of the ester is pH-dependent and depends on the basicity of the activator and the pH level reached. For a reliable temporal programming it is therefore important to use an alkaline buffer substance as activator, because this provides a jump to a well-controlled pH level.

Fig. 2a schematically shows the resultant pH loop (black curve) along with the expected response signal of the i-motif switch (blue curve). The IFS drives the temporary assembly of the i-motif switch by generation of a self-regulated, transient elevation of the ambient pH to the alkaline regime. The lifetime of the “on”-switched state can be programmed by controlling the molar ratio and chemical species of the chosen activator/DD pair.

With regard to the switching point of our pH-sensitive DNA-based system (*ca.* pH 6.0; Fig. 1c), we use tris(hydroxymethyl)aminomethane hydrochloride (Tris) of pH 8.8 as activator and β-butyrolactone (β-BL) as DD due to its ready water miscibility and steep pH decay profile upon hydrolysis. β-BL hydrolyzes in water to β-hydroxybutanoic acid (β-BA) with a p*K*_a_ value of 4.41, which is sufficiently acidic to protonate the i-motif switch (Fig. 2b).

**Fig. 3:**
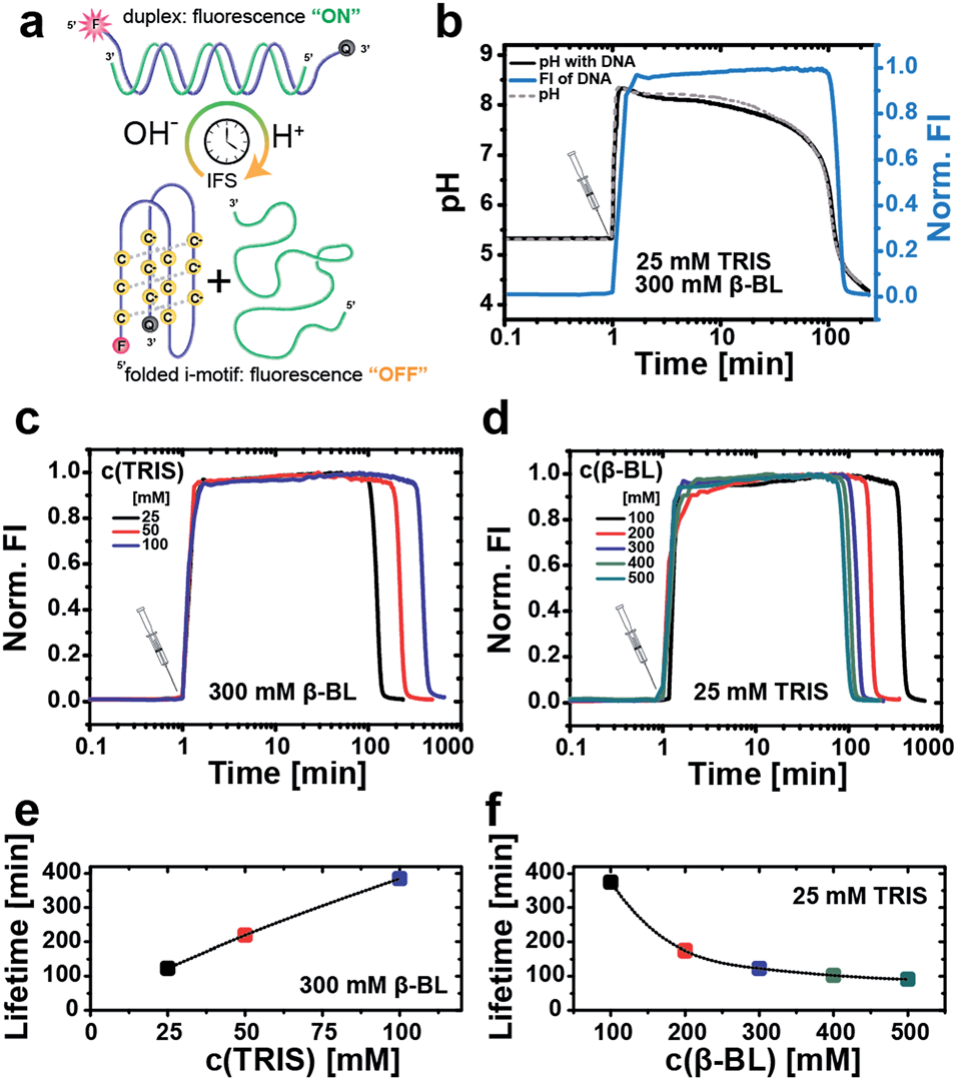
Autonomous operation of the i-motif switch with time-programmed transient fluorescence as output. (a) Schematic depiction of the IFS-driven fluorescent i-motif switch. (b) Generation of self-regulated transient fluorescence under dynamic pH control by an IFS of 25 mM Tris (activator) and 300 mM β-BL (DD). The fluorescence signal (blue line) is shown together with the pristine pH curve (dashed gray line) of the pure IFS, and with the internal pH of the system in presence of the i-motif switch (black line). Measurements are carried out in water with 0.5 μM DNA, 100 mM NaCl and 0.1 mM EDTA. All fluorescence traces are collected at *λ*_em,max_ = 564 nm (*λ*_exc_ = 520 nm) and normalized to maximum intensity. Temporal programming of the fluorescence signal by (c) variation of the activator Tris at a constant DD concentration of 300 mM β-BL, and (d) variation of β-BL (DD) at a constant activator concentration of 25 mM Tris. Corresponding lifetime plots of the fluorescence signal in dependence of (e) Tris (300 mM β-BL) and (f) β-BL (25 mM Tris).

Next, we first illustrate the operation principle of the time-programmed i-motif switch using the example of an IFS formed by 25 mM Tris and 300 mM β-BL (Fig. 3b). The starting pH of the system is set to pH 5.3, where the switch is inactive (“off”-state). Looking at the temporal evolution of the pH curve generated by the pristine IFS without the DNA i-motif (Fig. 3b, dashed gray curve), we identify the characteristic nonlinear behavior, which is needed for temporary activation of the pH-sensitive DNA switch. Injection of the IFS lifts the pH to 8.5 as a result of the immediate and fast allocation of the alkaline activator Tris. Afterwards, the pH initially decreases slowly because the released acid of the hydrolyzing β-BL is buffered by the activator Tris. After exhaustion of the activator/buffer capacity (*v*_act_ → 0), the pH drops promptly. The excess of the DD ensures the overall reduction of pH, closing the full loop from acidic to basic to acidic conditions.

In a next step, we couple this autonomously self-regulated pH loop to the fluorescently labeled i-motif switch. At this low i-motif switch concentration, the coupled pH curve (0.5 μM DNA, black) and the pH curve of the pristine IFS (non-coupled, dashed gray line) are almost overlapping, giving a lifetime of the transiently elevated pH milieu (pH ≥ 6) of 105 min and 102 min, respectively. We can use the fluorescence signal of the i-motif system to determine the lifetime of the duplex (“on”-state). The lifetime is defined to the range where the fluorescence intensity reaches 50% of the maximum value. For this IFS, the lifetime is just about 2 h (122 min). Interestingly, the fluorescence decay is delayed compared to the drop in the pH. We associate this slow kinetics of switching to the cooperative protonation mechanism of the i-motif folding, a potentially stabilized dehybridization at a slow pH decay (protonation using IFS decay) and to counterion effects of the IFS (different to classical HCl/NaOH switching). This kinetic delay needs to be considered for programming the lifetimes, but is not detrimental for reproducible temporal control.

Different lifetimes can be programmed by managing the interplay of the buffer capacity of the activator Tris with the concentration of the DD. For instance, the lifetime of the fluorescent “on”-state can be elongated by raising the molar ratio of the activator in the IFS. Fig. 3c and e summarize the obtained lifetimes upon increasing the Tris concentration from 25 mM to 50 mM and 100 mM at constant 300 mM β-BL. This extends the “on”-state from 2 h up to *ca.* 6.5 h. Similarly, we can expand the lifetime of the closed switch from 1.5 h to more than 6 h by reducing the DD concentration stepwise from 500 mM to 100 mM β-BL at an unchanged activator concentration of 25 mM Tris (Fig. 3d and f).

In summary, these results demonstrate that we are able to program for the first time the lifetime of a DNA duplex hybridization based on the i-motif DNA switch.

### Temporal programming of transient DNA–Au-NP networks

To progress towards time-programmed self-assembling DNA entities and autonomously dynamic, transient, hybrid materials, we capitalize on the developed concept by transducing the molecular control mechanism to a larger length scale, and demonstrate autonomous control over the self-assembly of gold nanoparticles (Au-NPs) and thus, control over plasmonic states in time on a functional level.

To this end, the two oligonucleotide sequences of the i-motif switch are extended with a spacer segment of nine thymines and a thiol group at the 3′-end. Starting from citrate-stabilized Au-NPs with a diameter of 20 nm, we prepared two separate batches of DNA–Au-NP conjugates, one decorated with the i-motif strand (**HS-I**) and another one with the complementary strand (**HS-C**). The Au-NPs are functionalized with those DNA binding motif strands, and, additionally, with short, non-hybridizing thiolated poly(thymine) **HS-T_9_** strands in a molar ratio of 1 : 9, respectively. Co-functionalization with this second strand enhances the colloidal stability of the single NPs and dilutes the surface grafting density of the DNA binding motif. This strategy improves the reversibility of the pH-dependent particle aggregation notably.

**Fig. 4:**
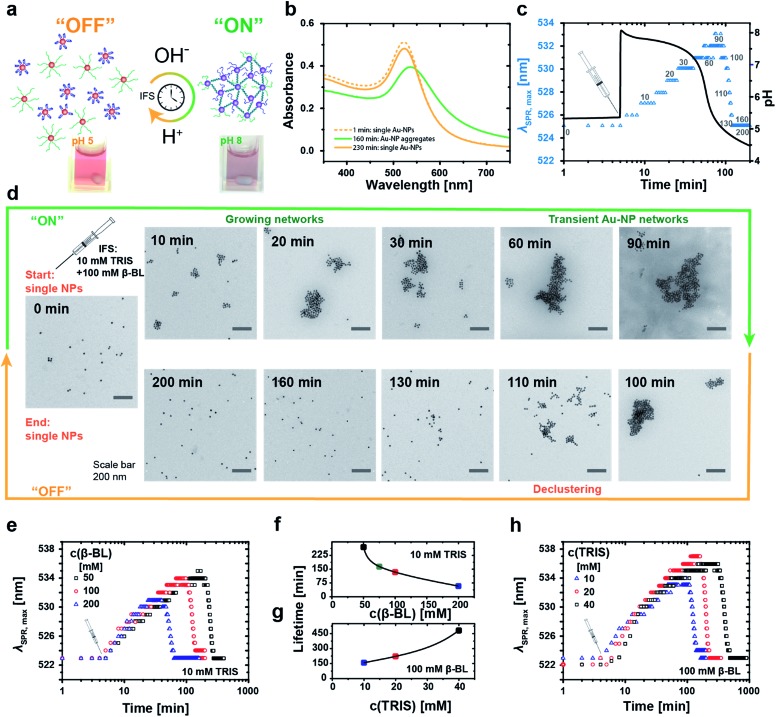
Temporal programming of transient DNA–Au-NP networks with limited lifetimes. (a) Transient co-assembly of Au-NPs, one carrying the i-motif and one carrying the complementary strand, mediated by the i-motif switch upon coupling to a pH-IFS. The aggregation-induced plasmon band shift serves as an optical tracer for the degree of assembly. (b) Representative UV-VIS spectra of the self-regulated aggregation of Au-NPs driven by an IFS of 20 mM Tris and 100 mM β-BL, showing the fully reversible time-programmed red-shift of the plasmon band during the “on”/assembly state. (c) Transient aggregation of Au-NPs controlled in time by an IFS of 10 mM Tris and 100 mM β-BL. The IFS is injected 5 min after mixing the Au-NPs in a 2 : 1 ratio of i-motif functionalized over complementary Au-NPs at pH 5.3 (10 mM Tris, 300 mM NaCl, 0.1 mM EDTA). Time-resolved UV-VIS trace of the maximum position of the plasmon band (blue triangles) along with the internal pH curve (black line) indicates a lifetime of the Au-NP aggregates of 130 min. (d) Aliquots at defined time intervals, highlighted by gray numbers in graph (c), were imaged by TEM to reveal the temporary assembly. (e–h) Lifetimes can be tuned by variation of the activator/DD ratio of the IFS: (e) reduction of the DD β-BL, or (h) increase of the activator Tris elongate the lifetimes of the aggregated state. Resulting lifetimes as a function of (f) β-BL at constant 10 mM Tris, and (g) Tris at constant 100 mM β-BL.

A stable red hydrosol of well-dispersed Au-NPs (Fig. 4a, left, pH 5) is maintained upon mixing both types of DNA-coated Au-NPs at slightly acidic pH 5, while aggregates occur upon setting to pH 8 (Fig. 4a, right, pH 8). This goes along with a strong shift in the surface plasmon resonance (SPR) frequency due to coupling of the plasmon modes in the assembled state. The pH-induced aggregation is solely mediated by the specific duplex hybridization of the active i-motif switch, as confirmed by a reference experiment, where defined satellite-core structures are obtained for co-assembling particle mixtures of different size (Fig. SI1†).

For temporal programming of the Au-NP co-assemblies, we intentionally added a two-fold excess of the i-motif functionalized Au-NPs to the binary mixture to limit the growth of the aggregates. This avoids precipitation during the time-dependent experiments, while still having a clear temporary change of the plasmonic properties. Time-dependent absorption spectra reveal the fully reversible IFS-driven growth of Au-NP networks (Fig. 4b). The plasmon band shifts gradually from 523 nm to *ca.* 540 nm, and broadens in the range of 550 nm and 700 nm due to hybridization-driven aggregation of the Au-NPs (Fig. 4b, green curve), before finally returning to the initial position and intensity (Fig. 4b, orange curve) of individually dispersed single Au-NPs.

A real-time tracking of the transient self-assembly process can be achieved by monitoring the position of the plasmon band (*λ*_SPR,max_) over time. Fig. 4c displays an exemplary system together with the corresponding pH curve. The injection of the IFS (10 mM Tris and 100 mM β-BL) activates the i-motif switch by the prompt increase to pH 8, and the isolated particles start assembling slowly and grow into larger networks. During this “on”-state, the plasmon band continuously shifts towards higher wavelengths. Meanwhile, the internal pH drops, and after approximately 90 min and returning to pH 5 the aggregates start to disassemble owing to the reprotonation of the cytosine bases with concurrent formation of the folded i-motif tetraplex, which unlocks the hybridized duplex linkages between the particles. The i-motif switch-driven NP assemblies are completely deactivated (“off”-state) after *ca.* 130 min as the plasmon band is relocated firmly at 523 nm. We define the duration of one such cycle as the lifetime of the Au-NP aggregates.

Fig. 4d displays the corresponding transient growth of Au-NP networks at defined time intervals *via* transmission electron microscopy (TEM). Increasingly larger cluster sizes can be observed till around 90 min, whereafter continuous disassembly follows.

The temporal fate of the aggregates depends on the chosen IFS and can be preprogrammed accordingly (Fig. 4e–h). For a constant activator amount of 10 mM Tris, successive reduction of the DD concentration from 200 mM to 50 mM β-BL leads to increased lifetimes from 1 h to 4.5 h (Fig. 4e and f).

Longer lifetimes can be targeted for higher activator concentrations. For instance, changing the ratio of Tris from 10 mM to 40 mM at a constant DD concentration of 100 mM β-BL gradually extends the lifetimes from 2.5 h up to 8 h (Fig. 4g and h). Importantly, the magnitude of the shift of the plasmon band increases with extended lifetimes due to a prolonged growth phase of the aggregates and larger aggregate sizes.

Consequently, the size of the aggregates and the optical properties of the Au-NP hydrosol are programmable by the timescale of the IFS. Overall, this coupling of the chemical reaction framework (IFS) with the molecular i-motif switch on the Au-NPs allows for active material properties by rational control of the time domain of the transient assembly, as transduced from a molecular level to a functional scale.

## Conclusions

In conclusion, we opened up a conceptual pathway to impart time-controlled dynamic features straightforwardly to synthetic DNA structures, and could for the first time demonstrate transient DNA duplexes with programmable lifetimes operating in an autonomous fashion. Our key approach is to couple the pH-responsive DNA i-motif switch with a universal temporal pH resetting mechanism, which is empowered by a simple chemical reaction framework. The autonomous behavior arises from the interplay of fast activators and slow dormant deactivators, which pushes the pH environment of the i-motif switch into a programmable transient alkaline pH state. We use this temporary “on”-switching of duplex hybridization to precisely program the lifetimes of fluorescence signals and Au-NP aggregation over several hours.

Thus, this approach permits the construction of dynamic and transient DNA structures under autonomous control without sophisticated sequence design of the oligonucleotides. Key advantages of the time-programmed i-motif switch are: (i) facile implementation into existing DNA systems and hybrid materials, (ii) fully autonomous operation in closed systems, (iii) broad tunability of the lifetime of the transient active duplex state by rational adjustment of the activator/DD ratio and by choosing other DDs.

With that in mind, autonomously dynamic, hierarchical DNA assemblies and DNA devices with time-programmed operation mechanisms and active material properties are in closer reach. Those may also involve other pH-sensitive DNA motifs *e.g.*, triplex or A-motif. We believe that adding time as an explicit design criterion will enhance the complexity of functional DNA assemblies and promote new types of applications by means of higher degrees of operational freedom and autonomy from outside triggers.

## Experimental section

Further materials and methods can be found in the ESI.†

### Temporal programming of the fluorescent i-motif switch *via* application of an internal pH feedback system

Samples were prepared from stock solutions of the oligonucleotides to give a concentration of 0.5 μM I* and C in a total volume of 150 μL containing 100 mM NaCl and 0.1 mM EDTA. The starting pH of the i-motif switch solution was kept at pH 5.3. After a short lag time, the internal feedback system (IFS) was added by simultaneous injection of the activator Tris pH 8.8 (from 2 M Tris stock *e.g.*, for 25 mM, 1.9 μL) and the dormant deactivator β-BL (*e.g.*, for 300 mM, 3.66 μL). The lifetime of the activated fluorescent i-motif switch was programmed in time by variation of the concentration ratio of the added IFS : Tris was varied from 25 mM to 100 mM, whereas β-BL was changed between 100 mM and 500 mM. The concentrations of the IFS relate to the initial volume of the i-motif switch solution. Time-dependent fluorescence profiles (excitation = 520 nm; recorded emission wavelength = 564 nm; slit 1 nm; integration time 0.1 s; interval 10 s) were measured in triplicate, averaged and normalized to the maximum fluorescence intensity.

### Time-programmed aggregation of Au-NPs monitored by the temporal evolution of the position of the surface plasmon resonance maximum

Initially, the solutions of the i-motif and the complementary strand-decorated Au-NPs were photometrically adjusted to the same stock concentration. This concentration was kept constant throughout the following experiments. Before each experiment, Au-NP stock solutions were mixed freshly using a two-fold excess of the i-motif functionalized Au-NPs to give a final volume of 1 mL. The pH was set to pH 5.3 with 1 M HCl_(aq)_. Subsequently, time-dependent UV-VIS spectra of the stirred solutions were collected in time intervals of 1 min with a resolution of 0.5 nm. After 5 min, transient aggregation of the Au-NPs was induced by simultaneous injection of the IFS: the activator Tris was varied from 10 mM to 40 mM and the dormant deactivator β-BL from 50 mM to 200 mM. Finally, all spectra were analyzed with respect to the position and intensity of the band of maximum absorbance.
