# Supplementary material for: Temporal control of i-motif switch lifetimes for autonomous operation of transient DNA nanostructures
Source: Chem Sci. 2017 Mar 31;8(5):4100–7. doi: 10.1039/c7sc00646b (PMC5439531; doi:10.1039/c7sc00646b)
Supplement: Supplementary file 1 [file SC-008-C7SC00646B-s001.pdf]

Electronic Supplementary Material (ESI) for Chemical Science.
This journal is © The Royal Society of Chemistry 2017



# Supporting Information

# "Temporal Control of DNA i-motif Switch Lifetimes for Autonomous Operation of Transient DNA Nanostructures"

**Laura Heinen, Andreas Walther***

andreas.walther@makro.uni-freiburg.de

*Institute for Macromolecular Chemistry, Stefan-Meier-Str. 31, Albert-Ludwigs-University Freiburg, 79104 Freiburg, Germany.*

*& Freiburg Materials Research Center, Stefan-Meier-Str. 21, Albert-Ludwigs-University Freiburg, 79104 Freiburg, Germany.*

*& Freiburg Center for Interactive Materials and Bioinspired Technologies, Georges-Köhler-Allee 105, Albert-Ludwigs-University Freiburg, 79110 Freiburg, Germany.*

## Table of Contents

1. Experimental Details ..... 2
   Materials ..... 2
   Au-NP Functionalization ..... 2
   Circular Dichroism ..... 3
   Fluorescence spectroscopy ..... 3
   UV-VIS spectroscopy ..... 3
   Transmission electron microscopy ..... 3
   Experimental Protocols ..... 4
2. Supporting Figure ..... 5

# 1. Experimental Details

## Materials

***Reagents:*** Ethylenediaminetetraacetic acid disodium salt dihydrate (EDTA, CALBIOCHEM, biology grade), 2-morpholinoethanesulfonic acid (MES, Sigma-Aldrich), sodium chloride (NaCl, 99%, ABCR), tris (hydroxymethyl)aminomethane hydrochloride (TRIS, Trizma Buffer substance pH 8.8, Sigma-Aldrich), β-butyrolactone (β-BL, 98% Sigma-Aldrich), tris(2-carboxyethyl)phosphine hydrochloride (TCEP HCl, Thermo Scientific), 1.0 M hydrochloric acid solution (Fluka), sodium hydroxide standard solution 0.999 N in $H_2O$ (Fluka), phosphate buffered saline (PBS, Sigma-Aldrich). 10 nm, 20 nm and 50 nm Citrate NanoXact Gold Nanospheres were purchased from nanoComposix.

***Oligonucleotides*** were purchased from Integrated DNA Technologies Inc. (HPLC purity). DNA stock solutions were prepared by resuspending the lyophilized oligonucleotides in water unless otherwise stated. Autoclaved Milli-Q water with conductivity less than 0.055 mS/cm was used throughout all experiments.

**Table 1: Oligonucleotide sequences**

| ID | oligonucleotide sequence |
| --- | --- |
| **i-motif strand (I)** | 5'-CCC TAA CCC TAA CCC TAA CCC-3' |
| **complementary strand (C)** | 5'-GTT AGT GTT AGT GTT AG-3' |
| **fluorescent i-motif strand (I*)** | 5'-/5Cy3/CCC TAA CCC TAA CCC TAA CCC/3BHQ_2/-3' |
| **thiolated i-motif strand (HS-I)** | 5'-CCC TAA CCC TAA CCC TAA CCC TTT TTT TTT/3ThioMC3-D/-3' |
| **thiolated complementary strand (HS-C)** | 5'-TGT TAG TGT TAG TGT TAG TTT TTT TTT/3ThioMC3-D/-3' |
| **dilution strand (HS-$T_9$)** | 5'-TTT TTT TTT/3ThioMC3-D/-3' |

## Au-NP Functionalization

The procedure for coating citrate-stabilized Au-NPs (20 nm) with thiolated oligonucleotides was adapted from Ref[1]. Two batches of Au-NPs were prepared: one functionalized with the thiolated i-motif strand (**HS-I**) and another with the complementary strand (**HS-C**) as DNA binding motif. The grafting density of the binding motif was diluted with a short **HS-$T_9$** strand in a molar ratio of 1:9 in feed. First, the lyophilized oligonucleotides **HS-I**, **HS-C** and **HS-$T_9$** were each dissolved in 10 mM PBS of pH 7.4 containing 0.1 mM EDTA. A total amount of 40 nmol (4 nmol DNA binding motif and 36 nmol dilution strand) of thiolated oligonucleotides was activated separately for each Au-NP batch by reduction for 30 min in freshly prepared disulfide cleavage buffer (10 mM PBS, 0.1 mM EDTA) with 0.01 M TCEP HCL as reducing agent. Subsequently, the activated oligonucleotide mixtures were each added to 10 mL of citrate-stabilized Au-NP hydrosol (nanoComposix, 0.05 g/L) and incubated for 1 h before the PBS concentration was adjusted to 9 mM by addition of 1 mL of phosphate adjustment

---

[1] Hill, H. D.; Mirkin, C. A., The bio-barcode assay for the detection of protein and nucleic acid targets using DTT-induced ligand exchange. *Nat. Protoc.* **2006,** *1* (1), 324-36.

buffer (100 mM PBS). After incubation for further 30 min, the salt concentration was increased stepwise to a final concentration of 300 mM NaCl by adding small aliquots of salting buffer (10 mM PBS, 2 M NaCl) every 30 min (6 aliquots in total). This salt ageing procedure was completed after 3 h. Then, the Au-NPs were allowed to equilibrate for 48 h and stirred at room temperature while being protected from light. Three cycles of centrifugation and redispersion in 10 mL of Au-NP storage buffer (10 mM TRIS, 0.1 mM EDTA, 300 mM NaCl) followed. Finally, DNA-functionalized Au-NPs were stored at 5 °C in the dark.

## Circular Dichroism

CD spectra were collected on a OLIS DSM 17 spectrometer in a 0.5 mm quartz glass cuvette with a volume of 140 µL from 350 nm to 200 nm using 150 increments and an integration time of 1 s. The chamber was continuously flushed with gaseous nitrogen. Samples contained 10 µM of each sequence of the i-motif switch (**I** and **C** respectively) without fluorophore labels and were measured in a buffer of 50 mM MES and 50 mM NaCl adjusted to either pH 5 or pH 8. All spectra were measured as triplicate, baseline corrected for the buffer blank and normalized to 350 nm.

## Fluorescence spectroscopy

Fluorescence measurements were performed on a Fluoromax-4 spectrofluorometer (HORIBA) using a cool-jacketed (20°C) ultra-micro fluorescence cuvette (Hellma) with a pathway of 3 mm. The concentration of the fluorescently labeled i-motif switch (equimolar amounts of **I*** and **C**) was kept constant at 0.5 µM throughout all fluorescent experiments.

## UV-VIS spectroscopy

UV-VIS spectra of Au-NP solutions were recorded on a Varian Cary 50 Bio UV/Visible Spectrophotometer in quartz glass cuvettes under continuous stirring against the Au-NP storage buffer solution (10 mM TRIS pH 8.8, 300 mM NaCl, 0.1 mM EDTA).

## Transmission electron microscopy

DNA-functionalized gold nanoparticles were imaged on hydrophilized carbon-coated copper grids in transmission mode on a Zeiss Libra TEM operated at 120 kV using zero-loss energy filtering.

## Experimental Protocols

***Determination of the transition midpoint of the i-motif switch:*** Various pH solutions (10 mM TRIS, 100 mM NaCl, 0.1 mM EDTA) in the range of pH 4 to pH 9 containing the fluorescent i-motif switch were prepared by adjusting to the respective pH with 1 M $NaOH_{(aq)}$ and 1 M $HCl_{(aq)}$ and equilibrated for at least 1 h. Full emission spectra were recorded in a range from 550 nm to 750 nm (increment 1 nm, integration time 0.1 s). The Cy3 fluorophore was excited at 520 nm. Fluorescence intensity was read at a wavelength of 564 nm and 605 nm for each pH solution and measured at least in duplicate, averaged and normalized to the overall maximum value. The sigmoidal fit of the data points in dependence of pH gave a transition midpoint of the fluorescent i-motif switch of pH 6.0.

***Multiple "ON/OFF"-switching of the i-motif switch:*** Repetitive cycling of the i-motif switch was shown in a standard quartz glass cuvette (pathway 10 mm) equipped with a stir bar. The fluorescent i-motif switch was dissolved in a buffer solution of 10 mM TRIS, 100 mM NaCl, 0.1 mM EDTA. Alternate addition of 1 M $HCl_{(aq)}$ and 1 M $NaOH_{(aq)}$ was applied to change between pH 4 and pH 8 repeatedly. After inducing the pH change, the solution was stirred shortly and equilibrated for 10 min before recording the emission spectrum. Fluorescence intensity ($\lambda_{exc}$ = 520 nm) was read at a wavelength of 564 nm and 605 nm for each cycling step and normalized to the maximum initial value.

***Time-programmed pH profiles of the pristine pH-IFS in aqueous solutions:*** Time-resolved pH profiles of the pure internal pH feedback system in aqueous solution were collected on a 12-channel pH-multimeter (EA Instruments LTD) with a measuring interval of 1 s while being stirred at 20 °C. Solutions contained 0.1 mM EDTA and in dependence of the corresponding self-assembly system either 100 mM NaCl (fluorescent i-motif switch) or 300 mM NaCl (Au-NPs). The IFS was added by simultaneous injection of the activator TRIS (from 2 M stock) and the dormant deactivator β-BL in the desired ratio (see description of the corresponding time-programmed self-assembly system for respective concentrations) to obtain time-programmed pH profiles with a transient alkaline pH level. Sample volume varied between 1 mL and 10 mL. All pH-profiles were measured in triplicate and averaged.

***Internal pH profiles corresponding to the time-programmed fluorescent i-motif switch:*** The internal pH of the time-programmed fluorescent i-motif switch solution was recorded in 2.0 mL at 20 °C using a pH multimeter (EA Instruments LTD) applying the same procedure and sample preparation as for the time-dependent fluorescence measurements (see experimental section of the manuscript). The pH-microelectrode was calibrated freshly on a daily basis.

***Internal pH profiles corresponding to the time-programmed aggregation of Au-NPs:*** The internal pH measurements of the time-programmed Au-NP aggregation were performed in 1 mL volume equipped with a stirring bar using a pH biotrode (Metrohm) applying the same procedure and sample preparation as for the time-dependent UV-VIS measurements of the Au-NP aggregation (see experimental section of the manuscript).

## 2. Supporting Figure

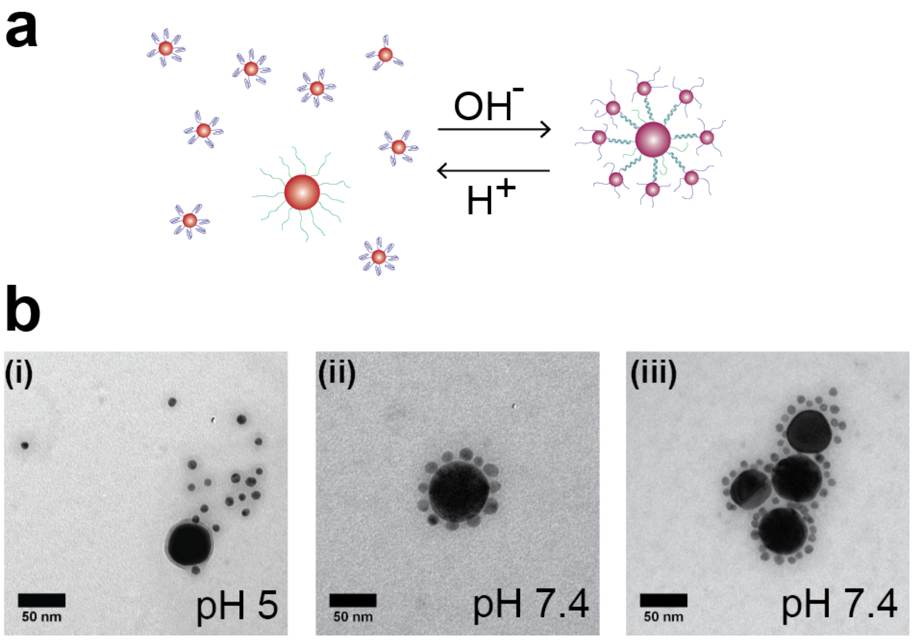


**Figure SI1: Specific, pH-dependent binding of i-motif switch-functionalized Au-NPs visualized by assembly of defined satellite-core structures. Satellite-core structures were assembled by mixing small 10 nm-sized Au-NPs carrying the i-motif sequence with larger 50 nm-sized Au-NPs functionalized with the complementary sequence in a volumetric ratio of 5:1. Functionalization was carried out according to the procedure described above. The pH was adjusted by $HCl_{(aq)}$. a) schematic drawing of the pH-dependent binding modes under acidic (left) and alkaline (right) conditions. b) TEM images from Au-NP solutions of pH 5 and pH 7.4 depicting the disassembled and assembled conformation of the DNA-Au-NP conjugates.**
